# Support Vector Machine Classification of Drunk Driving Behaviour

**DOI:** 10.3390/ijerph14010108

**Published:** 2017-01-23

**Authors:** Huiqin Chen, Lei Chen

**Affiliations:** 1 College of Mechanical Engineering, Hangzhou Dianzi University, Hangzhou 310018, China; chenlei@hdu.edu.cn; 2 State Key Laboratory of Advanced Design and Manufacturing for Vehicle Body, Hunan University, Changsha 410012, China

**Keywords:** drunk driving, support vector machine, principal component analysis, driving performance, physiological measurement

## Abstract

Alcohol is the root cause of numerous traffic accidents due to its pharmacological action on the human central nervous system. This study conducted a detection process to distinguish drunk driving from normal driving under simulated driving conditions. The classification was performed by a support vector machine (SVM) classifier trained to distinguish between these two classes by integrating both driving performance and physiological measurements. In addition, principal component analysis was conducted to rank the weights of the features. The standard deviation of R–R intervals (SDNN), the root mean square value of the difference of the adjacent R–R interval series (RMSSD), low frequency (LF), high frequency (HF), the ratio of the low and high frequencies (LF/HF), and average blink duration were the highest weighted features in the study. The results show that SVM classification can successfully distinguish drunk driving from normal driving with an accuracy of 70%. The driving performance data and the physiological measurements reported by this paper combined with air-alcohol concentration could be integrated using the support vector regression classification method to establish a better early warning model, thereby improving vehicle safety.

## 1. Introduction

As is well known, driving under the influence of alcohol is life-threatening. Data from both developed and developing countries show that the risks are similar. In Australia, a blood alcohol concentration (BAC) of 0.05% has been found in approximately 30% of all drivers fatally injured in crashes [1,2]. In Canada, 38.3% of the fatal driver injuries in 2003 were alcohol-related [2]. Drinking and driving is thought to be responsible for approximately 20% of all road fatalities in Europe every year [2,3]. In the U.S., alcohol-impaired driving was the cause of nearly 11,000 deaths—approximately one–third of all U.S. traffic-related fatalities in 2009 [4,5]. Approximately 34% of the road crashes in China were related to alcohol consumption [6], and Taiwan had 1973 fatal traffic accidents in 2010; of these, 373 were caused by drunk driving, resulting in 395 deaths [7].

Countries worldwide have enacted laws to prohibit driving after alcohol consumption that impose severe penalties on violators. The blood alcohol test is a popular way to detect drunk driving; it measures alcohol concentration in a driver’s blood. Blood alcohol levels can be measured with instruments from blood, urine, or exhaled breath. At present, the breath alcohol content detector is the most common testing mechanism used by Chinese authorities to judge whether a driver is drunk, and it is the only means by which law enforcement officials can determine levels of drunkenness on the spot. The principle behind the breath alcohol concentration detectors is similar to the principle used by vehicle-mounted alcohol concentration test devices in Sweden, Finland, and other countries [8,9], which are commonly referred to as alcohol lock or alcohol ignition interlock devices. This device was first developed by Volvo. After installation, a driver must first complete a breath test before driving; if the test results exceed a threshold, the car interlock device is activated and the driver will not be able to start the car. However, alcohol locks can be defeated; for example, a driver can get people who have not been drinking to take the test and, thus, unlock the ignition. To solve this problem, detection methods based on other physiological measurements and on driving performance have been proposed. 

Many studies have shown that physiological measurements, such as electroencephalography [10], can be used to distinguish drowsy drivers. Similarly, the differences between drunk drivers and normal drivers can be used to detect drunk driving by analyzing other physiological characteristics, such as heart rate variability [11]. Research into driving performance to explore the differences between drunk drivers and normal driver have also been carried out. Leung and Starmer [12] investigated the effects of a moderate dose of alcohol on a driver’s perception of speed, hazards, and risk acceptance; they concluded that young and mature drivers demonstrated pivotal differences in behaviour. Helland et al. [13] used ethanol as a positive control to examine how alcohol affects driving performances in a simulator and whether those effects were consistent with performances during real driving on a test track under the influence of alcohol. They found a positive dose-response relationship between higher ethanol concentrations and increases in the standard deviations of lateral position (SDLP) in both the simulator and on the test track. 

However, few studies exist that explore the integration of physiological measurements and driving performance to distinguish drunk driving from normal driving. Artificial neural networks (ANN), K-nearest neighbour (KNN), Bayesian networks (BN), and support vector machine (SVM) classifiers are quite popular. The SVM model is particularly effective for classification problems with small sample sizes. SVM models have been increasingly implemented in various transportation studies. In 2008, Li et al. [14] evaluated the efficiency of SVM models in predicting vehicle crashes. The results showed that SVMs were better than negative binomial models and back-propagation neural networks for crash prediction. Ren and Zhou [15] proposed a hybrid method that incorporated particle swarm optimization and SVM to make traffic safety forecasts. Robinel and Puzenat [16] used a multi-layer perceptron (MLP) and an SVM to determine whether a driver’s blood alcohol concentration (BAC) was above 0.4 g/L. Yu and Abdel-Aty [17] applied an SVM model to predict potential crashes in real time by considering actual traffic data ranging from 5 to 10 min before the crash occurrence. Chen et al. [18] employed SVM models to investigate the severity of driver injury patterns in rollover crashes based on two years of crash data gathered in New Mexico. Wang and Xi [19] used a rapid pattern-recognition method, called kMC-SVM, which developed by combining the k-means clustering and SVM, to recognize driver’s curve negotiating patterns. Li et al. [20] used an SVM classifier to classify drivers into two classes (normal or drunk) based on the extracted driving performance features. However, in this study, the driving performance and physiological measures were integrated together to classify the drunk drivers from normal drivers based on the SVM model. A disadvantage of the SVM model is that it lacks the capability to automatically select the significant factors that contribute to the target variable. Thus, principal component analysis (PCA) is often conducted to rank the variables by their relative importance and identify the most significant variables. This technique is increasingly used in the traffic field. Malagon-Borja and Fuentes [21] adopted PCA-based reconstruction to detect pedestrians. Nguyen and Kim [22] showed that using bidirectional PCA with vertical edge images was highly suitable for pedestrian detection. The PCA was also selected in the study of El Chliaoutakis et al. [23] to find some latent variables. 

The objective of this study was to predict whether drivers were drunk using both driving performance and physiological measurement data. The most previous algorithms have been based on only few eigenvalues, either the driving performance or the physiological signal, to make the determination of drunk driving. Therefore, they are prone to misjudge. The driving performance and physiological measurement data obtained in this study can be integrated by applying the support vector regression method to establish a better prediction model that can be used to prevent drunk driving.

## 2. Experiment Design

### 2.1. Participants

A total of 16 novice male drivers aged between 18 and 24 years and who had held a licence for no longer than 12 months were selected as the study participants. Most of the participants were recruited via paper flyers and the rest through recommendations.

### 2.2. Apparatus

Driving Simulator: The experiment was conducted in a portable driving simulator. The simulator runs on a PC platform under the Microsoft Windows operating system. Standard 3D graphic boards generate images. Drivers interact with the images through real Hyundai car controls. The steering wheel is linked to an active force feedback system controlled by the software based on the simulated speed of the car, the road surface, and the type of power steering. Moreover, a passive force feedback mechanisms incorporated into the pedals reproduce the feel of the clutch and brake from a real car.

The simulator is built around a dedicated PC that integrates the visual system, the sound system, and a custom I/O board. The image of the driving simulation is displayed on three LED screens. The driver is positioned approximately 0.8 m from the centre screen. The visual angle to the screens is 90 degrees, but the simulator software adjusts the image to provide a 120-degree field of view. The simulator sampling rate was 30 Hz.

Breath Alcohol Analyser: A breath alcohol analyser (Alcostop A, Justec Co., Ltd., Shenzhen, China) of the same type used by police authorities in China for routine roadside breath alcohol screening was employed in this study to measure the subjects’ breath alcohol levels. The resulting measurements were converted to BAC levels [24].

ErgoLab Human Machine Environment Synchronization System: Radio-frequency physiological recording technology, the behaviour code-analysis technology, and the human-machine environment synchronization technology was used in the ErgoLab human machine environment synchronization system, which achieves simultaneous recording, tracking and analysis of individual physiological, psychological, and behavioural factors. Electromyogram (EMG), electrodermal activity (EDA), and photo-plethysmography (PPG) wireless sensors were applied in this study. A Tobii eye tracker (Tobii, Danderyd, Sweden) with a sampling frequency of 60 Hz was employed to collect the participant’s eye movements, time of blink, and other visual measurements. Cameras were also used to collect driver behaviours.

### 2.3. Procedures

The participants were asked to obtain sufficient sleep the night before the experiment and refrain from consuming any food or drink that contained alcohol the day before the experiment. Two hours before the experiment, the participants were prohibited from consuming high-fat, high-sugar, or caffeinated substances that could affect alcohol absorption. The participants’ breath was analysed in the laboratory to ensure that their pre-test status was alcohol-free.

**Figure 1:**
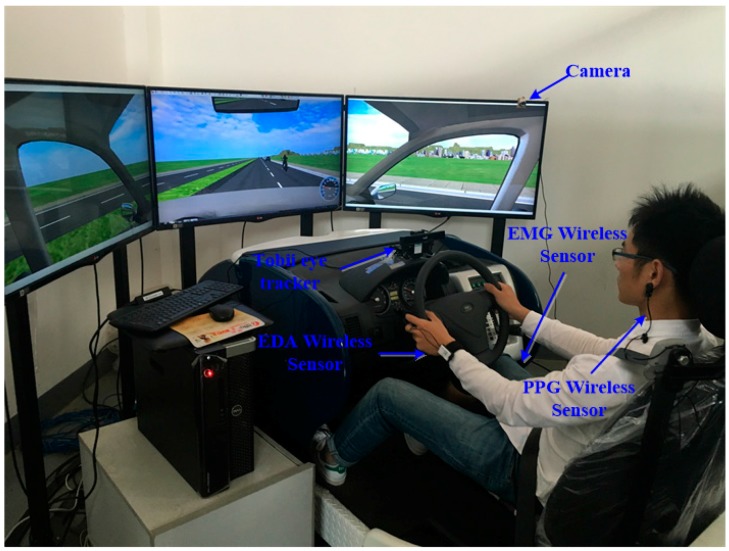
Participant in the simulator with sensors.

When the participants arrived in the simulation room, the laboratory staff introduced the basic processes: the driving task and the basic operation and safety information about the simulator. The participants signed consent forms officially consenting to participate in this experiment. The participants were then asked to provide demographic information such as gender, age and driving experience. The participants completed a current health questionnaire and were weighed. Then, the researcher explained the simulator study in detail. To familiarize participants with the simulator controls and dynamics, each participant practised driving in the simulator for 5 min. Eye tracker calibration was carried out before the practice session began. Then, the wireless sensors were put on. The ErgoLab physiological sensors (KingFar Technology Co., Ltd., Beijing, China) were applied to the participants as follows: the index and middle fingers were connected to the EDA sensors using finger buckles; the EMG sensors were fixed on the lower legs using bandages (to capture the electrical potential of leg muscles). The PPG sensor was attached to the earlobes to collect heartbeat signals. Finally, the Tobii eye tracker collected visual behaviour. Figure 1 shows an image of a participant in the simulator.

The participants were then given one of the following two alcohol treatments: a 0.0 g/kg (placebo group) or 1 g/kg (high dose group). The 1 g/kg treatment was intended to produce a target BAC of 80 mg/100 mL [24,25]. Eighty milligrams per 100 millilitres is the official level that indicates drunk driving in China.

The alcohol dose was calculated based on body weight and administered as pure alcohol mixed with orange juice. Based on the participants’ weight, the staff calculated the amount of alcohol and mixed it with twice that amount of orange juice. The participants drank the beverages within 15 min. The first BAC measurement occurred 10 min later, and the resulting BAC values were recorded. Participants in the placebo group received a 200-mL beverage that consisted of orange juice containing 3 mL of white wine added to the top of each drink. Their BAC values were also recorded. Of the 16 participants who completed the full experimental sessions, eight were assigned to the placebo group and eight to the high dose group.

The ascending BAC test was conducted 30 min after beverage consumption. The simulated road had two lanes in each direction, and the posted speed limit was 60 km/h. The participants finished the ascending BAC test in 15 min. The BAC descending test was conducted 90 min after beverage consumption. The simulator scene was counterbalanced to minimize the practice effects. Each subject’s BAC was measured at 10 min, 30 min (before and after the test), 60 min, 90 min (before and after the test), and 120 min after treatment administration. Physiological measurements were collected during the whole experiment time; in addition, the participants’ driving performances were recorded by the simulator.

After the testing was completed, the subjects remained at leisure in the lounge until their BACs fell to 20 mg/100 mL or below. Transportation home was provided after the sessions. Each participant was paid RMB 80 yuan for their participation.

### 2.4. Data Collection

To initialize the support vector machine model for prediction, the physiological measurements were collected and specific features were identified as follows:

Heart Activity: Heart rate can be monitored to assess the individual physiological level of workload. It has been shown that heart rate decreases significantly during a monotonous driving task [26]. Many studies have also validated the effectiveness of heart rate variability measures for diverse physiological conditions and the heart activity is a worthwhile research topic for further investigation [27]. A photoplethysmograph (PPG) signal was used in this study to obtain heart activity measurement. The following features are physiologically meaningful: the average heart rate (AVHR); the standard deviation of R–R intervals (SDNN), the root mean square of the difference between adjacent R–R interval series (RMSSD), and the percentages of the differences between adjacent R–R intervals greater than 50 ms (PNN50). The frequency domain measurements of the HRV power spectrum analysis were performed by fast Fourier transforms on specific time periods of the continuously recorded ECG RR intervals to obtain the frequency domain, including low frequency (LF: 0.04–0.15 Hz), high frequency (HF: 0.15–0.40 Hz), and the ratio of low frequency and high frequency (LF/HF).

Electrodermal Activity: Electrodermal activity (EDA) is frequently used as an indirect measure of attention, cognitive effort, or emotional arousal [27,28]. EDA can be distinguished into tonic and phasic parts. The skin conductance level (SCL) is the tonic value and shows the continuity of activity over time. The skin conductance response (SCR) is the phasic part and reveals changes in skin conductance within a short time period [27,29]. When individuals experience stress, the sympathetic nerves increase sweat secretion, resulting in increased EDA values. 

An electromyography (EMG) signal is associated with muscle contraction. EMG is the result of muscle excitation, which reflects the functional state of the muscle. EMG signals can be used to determine muscle fatigue and its degree. The following features are physiologically meaningful: root mean square amplitude (RMS), the average EMG (AEMG), the median frequency (Median Freq.), and the mean frequency (Mean Freq.). 

Several eye activity parameters have been shown to be sensitive to time on task, and research has shown that the disappearance of blinks, mini-blinks in eye movement, are the earliest reliable signs of drowsiness [27]. The eye activity monitoring could be used as an operator’s alertness level. The participants’ eye blink duration times were also analyzed in this study.

Finally, not only were physiological data collected, but the participants’ driving performances were also collected in this study. Speed is always used as an index to evaluate driver performance. Studies have shown that driving under influence offenders commit more moving violations, such as speeding, and are involved in more accidents compared with the general population [30,31]. Lane weaving is a common measure for assessing driving performance, and standard deviation of lateral position is a sensitive vehicular control indicator, often employed in drugged driving research [32,33]. Some studies have found that alcohol negatively affects behaviours such as steering wheel control and braking, as well, Fillmore et al. expressed that alcohol significantly impaired driving performance, which included deviation of lane position, line crossings, steering rate, and driving speed [2,34,35,36]. The statistical features of speed are maximum speed, minimum speed, mean speed. The standard deviation of steering wheel rotation angle and the standard deviation of lane position, where the lane position is the distance from the center of vehicle to the lane center, were also taken as features in this study. 

The description of the features are listed as the following Table 1. 

### 2.5. Ethical Satetment

All subjects gave their informed consent for inclusion before they participated in the study. The study was conducted in accordance with the Declaration of Helsinki, and the protocol was approved by the Ethics Committee of Third Xiangya Hospital, Central South University (No: 2016-S251). 

## 3. Methods

### 3.1. Principal Component Analysis

Principal component analysis is an effective method for statistically analyzing data. PCA is based on the Karhunen–Loève expansion [37], which has the advantage of greatly reducing the dimensions of feature data. The data are projected from the original high-dimensional space to a low-dimensional vector space using a special vector matrix. The low-dimensional vector contains those components with the largest variance that are not related to each other; consequently, an optimal feature extraction can be achieved. Although principal component analysis destroys the original feature space, the weight of the original feature in the principal component can be calculated. Then, the number of original features in the original feature space can be reduced. Feature selection based on PCA includes three parts: extraction of the co-principal components, calculation of the weights, and acquisition of the feature subset.

The degree to which each principal component can be generalized to a general feature is represented by its contribution rate; the principal component is a linear combination of the original features. Many principal components may be selected to represent the complete information of the entire original feature. Then, the weights of the original feature attributable to the principal components are obtained, which, together, equal the weight of the total characteristic. The larger the range of characteristic parameter variance is, the greater the weight is, and, consequently, the greater the impact a feature has on the results.

### 3.2. SVM and Kernel Function

The SVM model is a non-parametric method for solving classification problems based on statistical learning theory, and it is a kernel-based classifier. Since the SVM model has been well documented, the method is summarized only briefly below [18]. 

For training data consisting of N records that are linearly separable:

(1) $$(x_{1},y_{1}),\ldots ,(x_{i},y_{i}),\;i=1,2\ldots N$$

where *y_i_* is the class variable, *y_i_* = ±1, and *x_i_* ∈ *R^k^* represents a vector composed of *k* explanatory variables. Training an SVM model is a procedure that finds the best hyperplane such that training records with *y_i_* = ±1 are separated to either side of the hyperplane and the distances of the closest records to the hyperplane on either side are maximized. This maximization problem can be solved by introducing a Lagrange multiplier. A trained SVM classifier has the following basic form:

(2) $$f(x)=sign\left[\sum_{\forall i,\alpha_{i>0}}y_{i}\alpha_{i}(x_{i}\cdot x)+b\right]$$

where *α_i_* are the Lagrange multipliers, *x* is the support vector of the hyperplane used to classify records, *b* is a real number used to define the basic function of the hyperplane ω·*x* + *b* = 0, in which ω is a normal vector that is perpendicular to the hyperplane, and ω·*x* is the dot product of ω and *x*. For data that cannot be separated by a linear hyperplane, a non-linear transformation function Φ is needed to map the data into a higher dimensional space. A kernel function is applied for this non-linear transformation, defined as follows:

(3) $$k(x_{i}\cdot x_{j})=\Phi (x_{i})\cdot \Phi (x_{j})$$

Two major types of kernel functions have been developed and applied to SVM models: the inhomogeneous polynomial kernel function and the Gaussian radial basis kernel function (RBF), as defined below:

(4) $$K_{Poly}(x_{i}\cdot x_{j})={\left[(x_{i}\cdot x_{j})+1\right]}^{p}$$

(5) $$K_{Gaussian}(x_{i}\cdot x_{j})=\exp\left[-\gamma x_{i}-{x_{j}}^{2}\right]$$

The Gaussian radial basis kernel function was applied in this paper. In this kernel γ is the parameter that controls the kernel width.

## 4. Results

The driving performance data were collected from the driving simulator, and the physiological behaviour data were collected from the wireless sensors. In addition, eye blink data were collected from the Tobii eye tracker. For the driving performance and physiological measurements, 20 original features were selected that represent the time domain or the frequency domain characteristics of the various signals. The raw physiological index data were filtered by 50 Hz power frequency. Fast Fourier transforms were used to transform the data from the time domain to the frequency domain. PCA was used to analyze the 20 selected features and obtain the primary components. There were six principal components whose eigenvalue is greater than one, and their cumulative contribution to the total variance is 81.098%, as listed in Table 2. Other components whose eigenvalue is smaller than one were ignored in this study. 

A component matrix can be obtained based on principal components method. The top six principal components, without the specific meaning, extracted in Table 2 can be expressed as a linear combination of all features by the coefficient matrix as shown in Table 3. 

The normalized weight of each original feature can be calculated by the accumulation of the coefficient of each original feature of the principal component in Table 3 multiplied by the percent of variance in Table 2 and divided by the cumulative percent. The main original features—those with relatively higher weights—are listed in Table 4.

**Figure 2:**
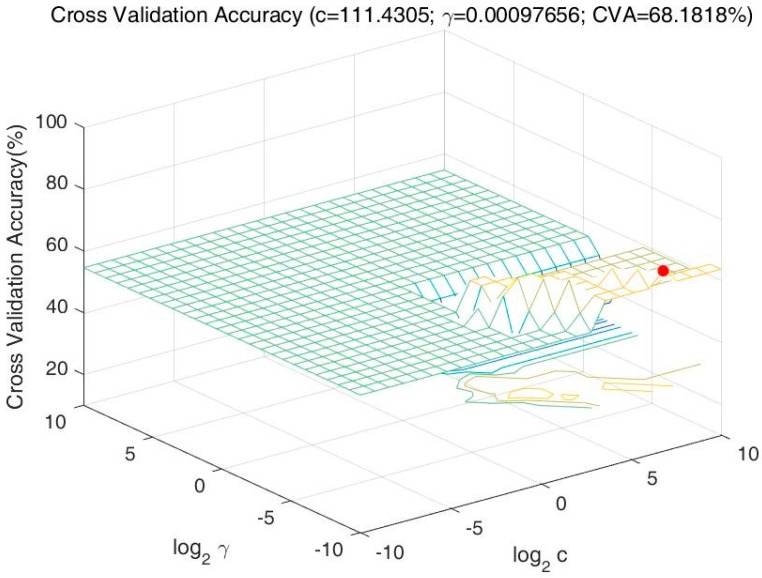
The relationship between the cross-validation accuracy and c, γ.

The experiments involved 16 participants and each participant underwent two trials, finally yielding 32 samples. Among these 32 samples, 22 samples, called the training set, were used to train the SVM model. The accuracy of the model in estimating the drunk condition was then assessed by the remaining 10 samples called testing set. SVM is considered as a black box, and the analytical equation usually found was not obtained here. The features selected were Z = (E_1_,E_2_,...,E_22_,E_2__0_)^T^. The expected outputs, y_i_ ∈ {−1,+1}, represent the classification of the driver’s drinking status (−1 = Drunk, +1 = Normal). After data normalization, the key parameters obtained after cross-validation: (c, γ) = (111.4305, 0.00097656), where log_2_c = 6.8 and log_2_γ = −10. The relationship between the cross-validation accuracy and c, γ is shown in Figure 2. For each pair of parameters to calculate the accuracy of cross validation, and the highest accuracy (68.1818%) presented as a red dot in the figure was obtained to determine the key parameters. Then the testing set was used to test the model’s classification accuracy, and the model’s accuracy rate is 70%. 

## 5. Discussion

Analyses of physiological measures have been included in previous studies in the field of traffic safety and are integrated with the driving performance analysis performed by this study. Although some studies exist concerning physiological measurements of drinking drivers and others have studied individuals’ driving performances, studies that integrate the two are rare. The EMG, EDA, PPG, and blink frequency reflect the fatigue, emotional arousal, and stress levels of drivers while driving. Moreover, the speed, the standard deviation of lane position and the standard deviation of steering wheel rotation angle can reflect the driver’s level of control over the vehicle. Here, 20 features were extracted from drivers’ physiological and driving performance measurements. PCA was used to obtain the feature weights and SVM was used to learn a classification model for drunk driving. 

The physiological signals includes features from RMS, AEMG, Median Freq, Mean Freq, Tonic Signal, Phasic Signal, SC, AVHR, SDNN, RMSSD, PNN50, LF, HF, LF/HF, and average blink duration, while the driving performance measurements included maximum speed, minimum speed, mean speed, standard deviation of lane position and standard deviation of steering wheel rotation angle. The simulated driving scenario was a straight road with two lanes in each direction for which the posted speed limit was 60 km/h.

The PCA results show that the weights of the original features have different impacts on the overall measurements. A relatively larger weight means the sensitivity of the feature is relatively larger when judging whether a driver is exhibiting drunk driving behaviour, and those features are more effective in accurately identifying drunk driving. SDNN, RMSSD, LF, HF, LF/HF, and average blink duration were the highest-weighted features in the study. SDNN index, which reflects the slow change of heart rate, is a sensitive index for evaluating the function of the sympathetic nerve. The RMSSD index reflects rapid changes in heart rate, which is a sensitive index to evaluate the function of the parasympathetic nerve. Its value decreases when the parasympathetic tone decreases. R–R time intervals were also extracted as the characteristic features for arrhythmia detection in Yu and Chou’s study [38]. Existing literature has shown that the high-frequency power of heart rate variability is an index associated with parasympathetic activity, and quite sensitive to the frequency and depth of respiration. The low-frequency band power of heart rate variability is an index associated with sympathetic activity. Thus, the LF/HF ratio can be considered as an index to assess the sympatho-vagal balance [39]. When a person is under stress, the LF band power and the LF/HF ratio increase. In addition, the LF power increases while the driving time increases, and the LF power decreases as the time of rest increases [40]. These data were all extracted from the PPG signal, indicating the importance of collecting heart activity data in the detection of drunk driving. Average blink duration can be used to determine a person’s arousal level. Many previous studies on eye activity have shown that sustained attention to a monotonous task may lead to performance fluctuations and eye activity changes. The relatively poor eye performances, such as longer blink duration, are generated with the fatigue and drowsiness [41,42]. 

In this study, the SVM model was trained to distinguish drunk driving and normal driving by integrating both driving performance and physiological measure data. Although the PCA was conducted to rank the highest-weighted features, the 20 features were still all in the SVM model. Speed is always chosen as an index to evaluate driver performance. Drivers are likely to speed when they have imbibed large amounts of alcohol. Motor-impairing effects of alcohol can reduce driver precision, resulting in greater within-lane swerving and line crossings. The disinhibiting effects of alcohol can compromise driving performance by increasing reckless behaviours, such as speeding, excessive lane changing, and a disregard of traffic signals [30]. Alcohol-impaired drivers can be slower to adjust the position of their vehicles in the road, a task that requires drivers to execute quick, abrupt steering wheel movements. Moreover, a driver’s steering wheel manipulations may change when drunk. These changes are reflected by an increase in steering wheel rotation angle. Driving performance, such as steering and braking control, is adversely affected by alcohol and previous studies indicated that the driver’s ability to control the steering wheel is seriously affected with a medium BAC level [2]. Steering wheel movement can be used to analyze the lateral control of the vehicle [43]. Another interesting indicator is the ability of the driver to position their car on the road in terms of lateral position (through its standard deviation) [44]. When drivers are drunk, their attention is scattered, and their reactions are slow. Consequently, they may fail to keep their vehicles within the proper lane. In the data, this manifests as a greater offset or greater standard deviation of lane position, indicating a poorer driving performance. Alcohol consumption reliably produces impairments in some behavioural measures, such as lane keeping, the number of centerline crossings and, particularly, the standard deviation of lane position [25,45]. These data were important inputs for the training model. 

There were 32 samples in the study, 22 were used to train the SVM model. The accuracy of the model in estimating the drunk condition was then assessed by the remaining 10 samples. The SVM results showed that the classification can successfully distinguish drunk driving from normal driving with an accuracy of 70%. Furthermore, this accuracy level could be improved by integrating detection of alcohol in the air. The main limitation of this study is its relatively small sample size, which undermines its statistical power and results in relatively lower prediction accuracy. However, the sample size is statistically sufficient to set up an SVM model, and the prediction accuracy may improve in future studies with larger sample sizes. University students are over-represented in the samples used in this study, and mature drivers would be studied in future studies. Since this SVM was developed as an early warning drunk driving detector, the trained SVM could be used as an automatic drunk driving detection method. 

## 6. Conclusions

Alcohol’s effect on cognitive and neurological functions is well-known and may create a high probability of traffic accidents. Studies on driver behaviour after drinking would provide theoretical support for the development of alcohol-related active safety system. This study conducted the detection experiment to distinguish drunk driving from normal driving under simulated driving conditions. The classification was performed by the support vector machine (SVM) classifier trained to distinguish between these two classes by integrating both driving performance and physiological measurements. In addition, principal component analysis was conducted to rank the weights of the features. The standard deviation of R–R intervals (SDNN), the root mean square value of the difference of the adjacent R–R interval series (RMSSD), low frequency (LF), high frequency (HF), the ratio of the low and high frequencies (LF/HF), and average duration time of blink were the highest weighted features in the study. SDNN, RMSSD, LF, HF, and LF/HF were all extracted from the PPG signal, indicating the importance of collecting HRV data in the detection of drunk driving. Average blink duration explains the importance of eye tracking in the study of drunk driving prevention. The physiological signals extracted features from EMG, EDA and PPG signals which including RMS, AEMG, Median Freq., Mean Freq., tonic signal, phasic signal, SC, AVHR, SDNN, RMSSD, PNN50, LF, HF, LF/HF, and average blink duration, as well, while the driving performance measurements included maximum speed, minimum speed, mean speed, standard deviation of lane position, and standard deviation of steering wheel rotation angle. All the physiological features and driving performance were integrated to train a SVM model. The results show that SVM classification can successfully distinguish drunk driving from normal driving with an accuracy of 70%. The prediction accuracy may improve in future studies with larger sample sizes. The driving performance data and the physiological measurements reported by this paper combined with air-alcohol concentration could be integrated using the support vector regression classification method to establish a better early warning model, thereby improving vehicle safety.

## Figures and Tables

**Table 1:** Description of the features.

Original Features	Explanation
AVHR	The average heart rate of photo-plethysmography signal
SDNN	The standard deviation of R–R intervals of PPG signal
RMSSD	The root mean square of the difference between adjacent R–R interval series
PNN50	The percentages of the differences between adjacent R–R intervals greater than 50 ms
LF	The low frequency of PPG signal
HF	The high frequency of PPG signal
LF/HF	The ratio of low frequency and high frequency
Tonic Signal, SCL	The tonic component of electrodermal activity signal
Phasic Signal, SCR	The phasic component of EDA signal
SC	The skin conductance of EDA signal
RMS	The root mean square amplitude of electromyography signal
AEMG	The average value of EMG signal
Median Freq.	The median frequency of EMG signal
Mean Freq.	The mean frequency of EMG signal
Average blink duration	The average of blink duration time
Maximum speed	The maxmimum value of the speed
Minimum speed	The minimum value of the speed
Mean speed	The mean of the speed
Standard deviation of lane position	The standard deviation of lane position
Standard deviation of steering wheel rotation angle	The standard deviation of steering wheel rotation angle

PPG, photo-plethysmography; EDA, electrodermal activity; EMG, electromyography.

**Table 2:** Contribution of the principal components to the total variance.

Component	Initial Eigenvalues		
	Total	% of Variance	Cumulative %
1	4.959	24.793	24.793
2	3.407	17.034	41.827
3	2.937	14.686	56.513
4	2.217	11.085	67.598
5	1.587	7.936	75.534
6	1.113	5.564	81.098
7	0.861	4.305	85.403
8	0.708	3.538	88.941
9	0.483	2.415	91.356
10	0.428	2.14	93.496
11	0.409	2.047	95.543
12	0.314	1.57	97.113
13	0.209	1.043	98.156
14	0.164	0.82	98.976
15	0.11	0.552	99.527
16	0.058	0.288	99.816
17	0.022	0.109	99.924
18	0.014	0.07	99.995
19	0.001	0.005	100
20	0	0	100

**Table 3:** Component matrix.

Original Features	Component					
	1	2	3	4	5	6
AVHR	−0.437	0.122	0.34	0.315	0.108	0.61
SDNN	0.955	0.11	−0.074	0.102	−0.097	0.088
RMSSD	0.957	0.115	−0.083	0.108	−0.106	0.081
PNN50	0.342	−0.069	−0.662	−0.007	−0.257	−0.138
LF	0.914	0.212	0.143	0.142	0.144	−0.065
HF	0.874	0.161	0.157	0.075	0.123	0.112
LF/HF	0.934	0.194	0.085	0.088	0.038	−0.017
Tonic Signal, SCL	−0.209	0.648	0.473	0.065	−0.279	−0.157
Phasic Signal, SCR	−0.226	0.646	0.24	0.299	-0.4	−0.067
SC	−0.248	0.739	0.411	0.204	−0.386	−0.13
RMS	−0.127	−0.466	0.249	0.6	0.229	0.101
AEMG	−0.112	−0.14	−0.152	0.66	0.234	0.17
Median Freq.	−0.115	0.602	−0.156	−0.53	0.371	0.235
Mean Freq.	−0.066	0.651	−0.109	−0.586	0.326	0.193
Average blink duration	0.207	0.357	0.694	0.197	0.362	−0.02
Maximum speed	0.019	−0.152	0.525	−0.28	0.488	−0.449
Minimum speed	0.219	−0.326	0.322	−0.444	−0.544	0.329
Mean speed	0.142	−0.505	0.514	−0.377	−0.216	0.091
Standard deviation of lane position	0.043	−0.477	0.577	−0.117	−0.032	−0.294
Standard deviation of steering wheel rotation angle	0.186	−0.327	0.538	−0.19	−0.018	0.328

**Table 4:** Normalized weight of original feature.

Original Feature	Normalized Weight
SDNN	0.120428528
RMSSD	0.120232149
LF	0.146177082
HF	0.138663629
LF/HF	0.137446247
Average blink duration	0.125356403
